# Stress and burnout in the context of workplace psychosocial factors among mental health professionals during the later waves of the COVID-19 pandemic in Hungary

**DOI:** 10.3389/fpsyt.2024.1354612

**Published:** 2024-03-27

**Authors:** László Molnár, Ágnes Zana, Adrienne Stauder

**Affiliations:** ^1^ Doctoral School of Semmelweis University Budapest, Budapest, Hungary; ^2^ Institute of Behavioural Sciences, Semmelweis University Budapest, Budapest, Hungary

**Keywords:** COVID-19, psychiatry, mental health, nurses, burnout, workplace stress

## Abstract

**Background:**

While literature is abundant on the negative mental health impact of the COVID-19 outbreak, few studies focus on the Central and Eastern European region.

**Objectives:**

We examined stress, burnout, and sleeping troubles among mental health professionals in the context of psychosocial risk factors related to participation in COVID care during the fourth and fifth waves.

**Materials and methods:**

Mental health professionals (N=268) completed an online cross-sectional survey in Hungary, between November 2021 and April 2022. Of the respondents, 58.2% directly participated in COVID care. The main data collection instrument was the Copenhagen Psychosocial Questionnaire (COPSOQ II), including 20 subscales on work-related psychosocial factors and 3 outcome scales (stress, burnout, and sleeping troubles). We added a question on competence transgression, and items on sociodemographic and professional background.

**Results:**

Participation in COVID care was associated with higher work pace (59.08 versus 49.78), more role conflicts (55.21 versus 45.93), lower scores on the influence at work (38.18 versus 51.79), predictability (44.71 versus 57.03), reward (55.82 versus 65.03), role clarity (70.19 versus 75.37), social support from supervisor (59.24 versus 65.55), job satisfaction (54.36 versus 62.84), trust regarding management (55.89 versus 67.86), justice and respect (44.51 versus 54.35) scales. Among those involved in COVID care, only the stress score was higher (47.96 vs. 42.35) in the total sample; however, among psychiatrists, both stress (52.16 vs. 38.60) and burnout scores (58.30 vs. 47.06) were higher. Stepwise multiple regression revealed that work-family conflict, emotional demands and workplace commitment were independent predictors of higher stress and burnout scores; furthermore, competence transgression had a significant effect on stress, and being a psychiatric specialist had a significant effect on burnout. These models explained 40.5% of the variance for stress and 39.8% for burnout.

**Conclusion:**

During the fourth and fifth waves, although COVID care was more well-organized, psychiatrists, as specialist physicians responsible for the quality of the care, were still experiencing challenges regarding their competence and influence at work, which may explain their increased levels of stress and burnout.

## Introduction

A large body of evidence demonstrates the negative impact COVID-19 exerted over the mental health of health care providers, including physicians, nurses, and allied health care workers. A meta-review published by Chutiyama et al. summarized the results of 40 systematic reviews, including 1828 primary studies, based on data from over 3,200,000 respondents. The authors concluded that increased stress and anxiety levels, burnout, sleep disorders, and various stress-related mental health problems signified a global issue, exhibiting some regional variation (1). However, there is a dearth of data from the Central-Eastern European (CEE) region, Poland connoting an exception (2).

No Hungarian study was included in Chutiyami et al.’s meta-review, despite the fact that Hungary suffered from one of the highest COVID-related mortality rates (3). Although during the first wave of the epidemic there was no significant excess mortality in Hungary (4), the second wave affected the country severely, and by the third wave, Hungary exhibited one of the worst mortality rates in Europe (5).

Merely two Hungarian studies were conducted on the mental health of health care workers (HCW) during COVID, one of which analyzed the attitudes and initial reactions of psychiatric ward workers in Budapest at the outbreak of the pandemic. The authors found that psychiatrists/psychologists, who had more knowledge about the status of the virus compared to nurses/other professionals, were more likely to experience higher levels of anxiety (6). The other study (7) scrutinized data from over 2000 HCW collected during the third wave of COVID (January-March 2021), when mortality rates were at their highest. They concluded that “COVID-19-related objective factors did not predict directly stress, burnout, and depression, whereas feelings of insecurity and unpredictability in relation to the COVID-19 situation at work had a significant medium-sized total effect (also considering the indirect effect via stress) on burnout and depression”.

The severity of the symptoms and the mortality rate attributable to coronavirus variants varied greatly over the 2020-2022 pandemic. These changes also connoted different working conditions for mental health workers, and congruently, altered personal perceptions and emotional reactions. Although COVID anxiety had significantly decreased, psychological distress, work exhaustion, perceived loneliness, and social support indicators did not change substantially (8).

The pandemic drew the public’s attention to the importance of promoting the mental health and well-being of HCW (9). Professionals working in mental health services experienced significant stress during the pandemic (10), and close contact with COVID-19 patients resulted in higher levels of anxiety and depression among mental health care workers (11). Mental health professionals who supported frontline workers also reported increased anxiety and increased workload (12). Alghamdi and colleagues found that the mean stress score of health care workers was even higher than individuals of the general population whom had been in contact with COVID-infected patients (13, 14). The main sources of stress among health care professionals were: lack of effective COVID-19 treatment, worry for their families’ and their own health, uncertainty in most areas of daily and professional life related to the rapid spread and the high mortality of the COVID-19 virus, and lack of preparedness to manage the crisis caused by the pandemic (14–16). One of the most frequently analyzed indicators in studies during the pandemic was burnout among health workers. An Australian study reported high levels of burnout among mental health professionals during the pandemic (burnout scores indicated moderate or higher workplace-related burnout for 40.6% of respondents) (17). Other studies focused on the psychosocial factors predicting high burnout. According to Gimenez-Espert and colleagues, the most prominent psychosocial risks appeared to be emotional work and workload (18), while Claponea et al. state that the most common psychosocial risks for health workers are workload, lack of organizational justice, emotionally demanding work, conflicting demands, and role conflicts (19). A systematic review and meta-analysis of 18,935 nurses found that during the pandemic, “the main risk factors that increased nurses’ burnout were the following: younger age, decreased social support, low family and colleagues readiness to cope with COVID-19 outbreak, increased perceived threat of COVID-19, longer working time in quarantine areas, working in a high-risk environment, working in hospitals with inadequate and insufficient material and human resources, increased workload and lower level of specialized training regarding COVID-19” (20). Several additional factors have been associated with burnout, such as shift work, increased job pressure, work-family conflict, and “practice environment satisfaction”, while salary satisfaction was a protective factor against burnout (21). According to Sklar et al., changes in working conditions resulting from COVID-19 care have led to increased turnover through burnout (22).

All related studies have a marked emphasis on the importance of offering tailored mental health support for affected health professionals on both the individual and the organizational level (1). As the World Psychiatric Association delegates this task to the psychiatrists themselves (23), the responsibilities of psychiatrists and mental health specialists substantially increased during the pandemic: besides providing for psychiatric patients and attending to COVID-related mental health problems in the general population, they were also expected to support HCW impacted by the emotional burden of COVID care.

Inevitably, COVID has severely impacted mental health care practitioners as well, both personally and professionally (24). Social distancing measures fundamentally altered mental health care delivery; psychiatric wards were closed or transformed to COVID care units, the hospitalization of psychiatric patients became very challenging. In tandem, telepsychiatry developed and disseminated rapidly, revealing that online consultations and therapies were also efficacious (25). Crocker et al.’s review, based on 55 studies, most of them published between 2020 and 2021, stated that *“*Key work-related outcomes included increased workload, changed roles, burnout, decreased job satisfaction, telehealth challenges, difficulties with work-life balance, altered job performance, vicarious trauma and increased workplace violence. Personal outcomes included decreased well-being, increased psychological distress, and psychosocial difficulties. These outcomes differed between inpatient, outpatient, and remote settings” (26). In this review, only two studies from the CEE region were included, both reflecting the initial reactions, during the first wave of the pandemic (6, 27).

Another reason to focus on psychiatrists in the context of their increased workload during the pandemic is that according to the pre-pandemic literature, among physicians, psychiatrists are at a particularly high risk of burnout, stress, alcohol abuse, drug abuse, and suicide (28, 29), and may be more vulnerable to burnout than other medical professionals (29–32). High burnout among doctors working in psychiatric care has also been reported in other studies (e.g., 33–40).

The aim of our study was to evaluate work-related psychosocial risk factors in COVID care units in comparison to non-COVID care units among mental health professionals in Hungary. Measured mental health indicators were stress, burnout, and sleeping troubles; we aimed to analyze their associations with work-related psychosocial factors in the context of participation in COVID care. Drawing on the literature, we also examined potential differences in the risk profile of professional subgroups with the psychiatric team, with special focus on psychiatrists and nurses.

## Materials and methods

### Study design and sample

We conducted a cross-sectional survey during the pandemic between November 15, 2021 and April 15, 2022. The anonymous online questionnaire was made available via Google Forms. Our target group was mental health professionals working in psychiatric and psychotherapeutic care in Hungary.

### The data collection process

We employed “targeted sampling”, also known as purposive or judgmental sampling. We utilized online platforms of professional organizations: our call was published on the website and the newsletter of the Hungarian Psychiatric Society, the website of the Hungarian Chamber of Health Care Professionals, and the Facebook groups of psychologists and the Hungarian Association of Psychiatric Trainees (HAPT). In addition, we contacted most heads of psychiatric wards in Budapest and the rest of the country (by email or phone), and we also wrote letters to relevant hospital directors to encourage participation in the study. Furthermore, we reached out to psychiatric nurses/specialists, psychotherapy inpatient and outpatient services, as well as child- and adolescent psychiatric inpatient and outpatient services. During the data cleaning process, we controlled for duplicates; there were none. Exact response rates could not be calculated due to the sampling method; we could not calculate the exact number of persons reached, however, based on public statistical data, we estimate that approximately 10% of health care professionals working at psychiatry care units in Hungary responded to the survey.

### Measurements

As our main data collection instrument, we employed the Hungarian version of the Copenhagen Psychosocial Questionnaire (COPSOQ II) (41, 42). COPSOQ II was chosen as a validated questionnaire whose subscales cover the most relevant work-related psychosocial risk factors and include relevant outcome measures. It has been widely used internationally and has a validated Hungarian version. We included 20 subscales on psychosocial work environment in our analysis, 3 or 4 items each, and 3 outcome scales: stress, burnout, and sleeping troubles. In our sample, the internal consistency of the subscales was good or acceptable, ranging from 0.656 to 0.916 (see **Table 1**).

**Table 1:** Psychosocial factors according to COVID care.

Covid care	Scale internal consistency	Sample total (N=268)		COVID care (N=156)		Not in COVID care (N=112)		Between Groups ANOVA	
Psychosocial factors at work	Cronbach alfa	Mean	SD	Mean	SD	Mean	SD	F	Sig.
Demands quantitative	0.76	44.38	20.63	45.27	18.90	43.14	22.87	0.698	0.404
Work pace	0.846	55.19	22.29	59.08	20.00	49.78	24.21	11.816	0.001
Emotional demands	0.687	68.07	18.14	68.03	17.53	68.14	19.04	0.002	0.962
Influence at work	0.775	43.87	22.59	38.18	19.15	51.79	24.62	25.855	<0.001
Possibilities for development	0.656	72.99	17.40	71.55	16.29	75.00	18.74	2.570	0.110
Meaning of work	0.828	79.17	19.86	77.83	18.94	81.03	21.02	1.692	0.195
Workplace commitment	0.844	61.68	25.64	59.21	24.50	65.12	26.88	3.495	0.063
Predictability	0.748	49.86	25.66	44.71	24.01	57.03	26.25	15.867	<0.001
Reward	0.887	59.67	25.55	55.82	25.25	65.03	25.11	8.710	0.003
Role clarity	0.802	72.36	20.43	70.19	20.44	75.37	20.12	4.243	0.040
Role conflicts	0.715	51.33	19.69	55.21	17.85	45.93	20.90	15.268	<0.001
Quality of leadership	0.88	60.87	25.02	57.49	24.82	65.57	24.65	6.944	0.009
Social support from supervisor	0.844	61.88	25.00	59.24	26.09	65.55	23.02	4.201	0.041
Social support from colleagues	0.744	62.72	20.49	61.59	20.64	64.29	20.27	1.127	0.289
Social community at work	0.81	75.28	19.52	75.59	18.19	74.85	21.31	0.092	0.761
Job satisfaction	0.75	57.90	20.42	54.36	20.02	62.84	20.02	11.709	0.001
Work-family conflict	0.878	47.02	28.98	48.08	28.32	45.54	29.93	0.502	0.479
Trust regarding management	0.781	60.89	22.11	55.89	20.75	67.86	22.15	20.497	<0.001
Mutual trust between employees	0.74	63.31	20.77	62.29	19.43	64.73	22.51	0.904	0.343
Justice and respect	0.834	48.62	23.57	44.51	22.25	54.35	24.24	11.831	0.001
Burnout	0.916	56.09	24.08	57.81	23.13	53.68	25.26	1.923	0.167
Stress	0.896	45.62	22.04	47.96	21.73	42.35	22.14	4.263	0.040
Sleeping troubles	0.867	32.88	24.48	34.05	25.54	31.25	22.94	0.855	0.356
Competence transgression	single item	1.97	0.79	2.04	0.78	1.87	0.79	2.763	0.098

Additionally, we included an item on competence transgression: “In your opinion, to what extent are your overall professional competencies violated during your work?” The response options were: “to a great extent/to a moderate extent/to a small extent/not at all”.

Items on sociodemographic and professional background included sex, age, occupation, specialization, years of work experience in specialty, location of workplace (capital or countryside), and whether work was performed in COVID care.

### Statistical analysis

Analyses were conducted using the IBM SPSS 26.0 software package.

We used the Pearson correlation coefficient to determine the correlations between work-related psychosocial factors and stress and burnout. As a multivariate analysis, we conducted a linear regression analysis with the stepwise method to evaluate the independent effects of the relevant psychosocial factors.

### Ethical considerations

Our survey was approved by the Ethics Committee of the Semmelweis University (SE-TUKEB: 270-1/2017). Participants were informed about the purpose of the study and their participation was voluntary. We did not collect any personal identifier data and kept demographic data as general as possible (e.g., instead of year of birth, we used age ranges.). Participants gave their consent to take part in the study by completing the anonymous online questionnaire.

## Results

### Sample characteristics

A total of 268 Hungarian persons, 208 women (77.6%) and 60 men (22.4%) completed the online questionnaire. The characteristics of the sample are presented in **Table 2**.

**Table 2:** Demographic characteristics of the sample.

	Sample size	Participated in COVID care		Between group difference	
	Total N	N	%	Chi square	Exact Sig. (2-sided)
All respondents	268	156	58.21		
**Profession**				27.251	<0.001***
Psychiatrist	86	52	60.47		
Resident	37	29	78.38		
Psychologist	47	15	31.91		
Nurse	84	56	66.67		
Other	14	4	28.57		
**Sex**				7.27	0.007
Male	60	44	73.33		
Female	208	112	53.85		
**Age group**				8.822	0.266
<31	34	25	73.53		
31-35	48	27	56.25		
36-40	39	23	58.97		
41-45	32	14	43.75		
46-50	37	24	64.86		
51-55	24	12	50.00		
56-60	33	21	63.64		
>60	21	10	47.62		
**Workplace location**				2.399	0.121
Budapest	125	79	63.2		
Countryside	143	77	53.85		

Regarding occupation, there were 86 physicians (32% of the sample) retaining one or more specializations, 37 (13.8%) residents in their first to fifth year of specialization training in adult or child psychiatry, 47 psychologists (17.5%), 84 nurses (31.3%), and 14 persons with a degree classified as “other”. Since the latter group was deemed heterogeneous, we excluded them from occupation-based comparison. In terms of specializations: 75 were psychiatrists, 12 were child and adolescent psychiatrists, and 1 was classified as “other”. (Two of these specialists were also board certified in psychiatry and child psychiatry.) Hereafter we refer to these specialists collectively as psychiatrists. Among the physicians in training, 22 were residents (i.e., in the first two years of their training) and 15 were trainees (i.e., in the last three years of their training) in adult psychiatry or in child and adolescent psychiatry specialization training; hereafter we refer to them collectively as psychiatry residents. Among the nurses, 17 were qualified nurses, 52 were specialized nurses, and 15 were assistant nurses. As psychologists were a heterogeneous group, they were not subjected to further categorization.

Overall, 156 respondents (58.21%) participated in COVID care. There were significant group differences concerning occupation: 78% of psychiatry residents, 67% of nurses, and 60% of psychiatrists worked in COVID care, while this proportion was only around 30% for psychologists and “other” professionals. There was a comparable number of respondents working in Budapest (N=125, 46.6%) and in the countryside (N=143, 53.4%); we found no significant regional difference in their involvement in COVID care.

The comparison of mental health professionals involved in and those not involved in COVID care revealed several differences regarding work-related psychosocial factors (see **Table 1**). We found significantly higher mean scores for work pace (59.08 versus 49.78) and role conflicts (55.21 versus 45.93) among those working in COVID care. Those who participated in COVID care had significantly lower scores for influence at work (38.18 versus 51.79), predictability (44.71 versus 57.03), reward (55.82 versus 65.03), role clarity (70.19 versus 75.37), social support from supervisor (59.24 versus 65.55), job satisfaction (54.36 versus 62.84), trust regarding management (55.89 versus 67.86), as well as justice and respect (44.51 versus 54.35). Regarding mental health indicators, only the stress score was significantly higher among COVID care workers (47.96 ± 21.73 compared to 42.35 ± 22.14), while there were no significant differences in burnout and sleeping troubles scores.

As a next step, we compared the stress, burnout, and sleeping troubles scores of each professional group in the context of their participation in COVID care (see **Table 3**). Only psychiatry specialists exhibited significant differences in stress and burnout scores. Those involved in COVID care had a higher mean stress score (52.16 ± 22.41 versus 38.60 ± 19.55; p=0.005) and a higher mean burnout score (58.30 ± 23.54 vs. 47.06 ± 23.40; p=0.033) compared to those not involved in COVID care.

**Table 3:** Stress, burnout, and sleeping troubles in psychiatry care workers participating in and not participating in COVID care.

	Profession	COVID Care			ANOVA	
Means		No	Yes	Total	F	Sig.
**Stress**	Psychiatrist	38.60	52.16	46.80	8.306	0.005
	Resident	50.78	51.51	51.35	0.006	0.938
	Psychologist	46.68	42.92	45.48	0.327	0.570
	Nurse	41.74	43.97	43.23	0.203	0.654
	Total	42.95	48.11	46.04	3.443	0.065
	ANOVA between professions				1.245	0.294
**Burnout**	Psychiatrist	47.06	58.29	53.85	4.705	0.033
	Resident	64.84	56.47	58.28	1.025	0.318
	Psychologist	60.55	63.33	61.44	0.123	0.727
	Nurse	53.13	56.70	55.51	0.403	0.527
	Total	54.35	57.85	56.45	1.318	0.252
	ANOVA between professions				1.143	0.332
**Sleeping troubles**	Psychiatrist	29.60	33.41	31.90	0.497	0.483
	Resident	32.81	24.35	26.18	1.646	0.208
	Psychologist	24.61	26.67	25.27	0.087	0.769
	Nurse	41.07	41.96	41.67	0.022	0.882
	Total	31.43	34.17	33.07	0.774	0.380
	ANOVA between professions				6.597	<0.001

Mean scores based on the COPSOQ II questionnaire.

We also compared the professional groups to each other (**Table 3**) and found a significant difference (p=0.001) only in sleeping troubles: nurses scored higher (M=41.67, SD=25.73) compared to the other professional groups (psychiatrists: M=31.90, SD=24.48; psychiatry residents: M=26.18, SD=16.66; psychologists: M=25.27, SD=22.04). We found no difference in terms of burnout or stress.

A correlation analysis (**Table 4**) was conducted to investigate the relationship between psychosocial factors and stress/burnout. The strongest correlations were found with emotional demands and work-family conflict (emotional demands with stress: r=0.380, with burnout: r=0.376; while work-family conflict with stress: r=0.543, with burnout: r=0.540). Among psychosocial factors, workplace commitment negatively correlated with stress (r=-0.313, p<0.001) and burnout (r=-0.283, p<0.001). Influence at work exhibited a significant correlation with stress (r=-0.2, p=0.001) and burnout (r=-0.212, p<0.001), albeit a markedly weak negative correlation.

**Table 4:** Correlation between work-related psychosocial factors and mental health indicators (N=268).

	Stress		Burnout	
	Pearson Correlation (r)	Sign. (p)	Pearson Correlation (r)	Sign. (p)
Demands quantitative	0.269**	<0.001	0.292**	<0.001
Work pace	0.153*	0.012	0.171**	0.005
Emotional demands	0.380**	<0.001	0.376**	<0.001
Influence at work	-0.200**	0.001	-0.212**	<0.001
Possibilities for development	-0.149*	0.014	-0.097	0.113
Meaning of work	-0.235**	<0.001	-0.190**	0.002
Workplace commitment	-0.313**	<0.001	-0.283**	<0.001
Predictability	-0.250**	<0.001	-0.182**	0.003
Reward	-0.231**	<0.001	-0.177**	0.004
Role clarity	-0.172**	0.005	-0.144*	0.018
Role conflicts	0.275**	<0.001	.0198**	0.001
Quality of leadership	-0.190**	0.002	-0.182**	0.003
Social support from supervisor	-0.130*	0.033	-0.130*	0.033
Social support from colleagues	-0.172**	0.005	-0.092	0.132
Social community at work	-0.173**	0.004	-0.095	0.121
Job satisfaction	-0.228**	<0.001	-0.243**	<0.001
Work-family conflict	0.543**	<0.001	0.540**	<0.001
Trust regarding management	-0.132*	0.031	-0.075	0.221
Mutual trust between employers	-0.163**	0.008	-0.055	0.371
Justice and respect	-0.215**	<0.001	-0.183**	0.003

The strength of significance was expressed as follows: * p<0.05; ** p<0.01.

Lastly, employing multivariate analysis, we tested the predictors of stress and burnout (**Table 5**), including all psychosocial work factors: quantitative demands, work pace, influence at work, possibilities for development, meaning of work, predictability, reward, role clarity, role conflicts, quality of leadership, social support from supervisor, social support from colleagues, social community at work, job satisfaction, trust regarding management, mutual trust between employers, justice and respect, work-family conflict, emotional demands, workplace commitment, participation in COVID care, competence transgression, and professional background (nurse, psychologist, psychiatrist, psychiatry trainee, and psychiatry resident). Profession and competence transgression were included in the model as DUMMY variables (0-1 values). These variables were selected as potential factors, and in accordance with the algorithm, non-significant variables were selected out using the stepwise method. This model explained 40.5% of the variance for stress and 39.8% for burnout (adjusted R squares: stress 39.6%, burnout 38.9%).

Table 5Results of regression analysis for stress and burnout (N=268).

**Table:** 

Stress				
	B	Beta	t	p value
(Constant)	20.308		3.649	<0.001
Work-family conflict	0.32	0.421	8.079	<0.001
Workplace commitment	-0.2	-0.232	-4.712	<0.001
Emotional demands	0.244	0.201	3.878	<0.001
Competence transgression	3.039	0.108	2.181	0.03
R-square	0.405			
F test	44.79			
Sign. (p)	<0.001

**Table:** 

Burnout				
	B	Beta	t	p value
(Constant)	33.954		6.377	<0.001
Work-family conflict	0.373	0.449	8.554	<0.001
Workplace commitment	-0.209	-0.222	-4.607	<0.001
Emotional demands	0.294	0.222	4.265	<0.001
Psychiatrist	-7.959	-0.155	-3.187	0.002
R-square	0.398			
F test	43.524			
Sign. (p)	<0.001			

The variables excluded in the stepwise method are: demands quantitative, work pace, influence at work, possibilities for development, meaning of work, predictability, reward, role clarity, role conflicts, quality of leadership, social support from supervisor, social support from colleagues, social community at work, job satisfaction, trust regarding management, mutual trust between employers, justice and respect, COVID care, nurse, psychologist, psychiatrist, psychiatry trainee, resident, competence transgression.

Stress levels were significantly influenced linearly by work-family conflict, emotional demands, competence transgression, and workplace commitment. While the first three had a positive effect on stress, workplace commitment had a negative effect (i.e., the more committed one was to their job, the less stress one experienced). Competence transgression exhibited the strongest linear relationship with stress. The degree of burnout was significantly affected by work-family conflict, emotional demands, workplace commitment, and by being a psychiatry specialist. The last two variables were negatively associated with burnout (i.e., being committed to one’s job and being a psychiatrist were associated with lower burnout scores). All other variables related to profession were deselected in the stepwise method.

## Discussion

We compared work-related psychosocial factors among mental health professionals involved in and those not involved in COVID care during the fourth and fifth waves of the COVID-19 pandemic in Hungary. Similar to what other studies have shown, those involved in COVID care scored their work environment as necessitating a higher work pace and instigating more role conflicts, being less predictable, having less influence on their actual work, less justice and respect, yielding less rewards, and also having less trust regarding management and being less satisfied with the leadership (**Table 1**). Prior to the pandemic, a number of studies had already investigated the stress and burnout levels of mental health professionals and their relationship to psychosocial factors. In this context, Rössler brought attention to certain job characteristics, such as workload, lack of social support (especially from supervisors/managers), lack of adequate information, role ambiguity or role conflict, and restricted autonomy (43).

Despite the prominence of these psychosocial risk factors, we found that in the case of psychiatric workers participating in COVID care, they were associated only with slightly elevated stress levels; however, no significant difference was found in terms of burnout. Thus, our study indicates that participation in COVID care does not in itself cause burnout. This was an unexpected result, as according to Chutiyami’s meta-review (1) and a scoping review by Moitra et al. (44), frontline work is generally a risk factor for stress and burnout among health care workers.

During the first three waves of the pandemic, without effective treatment or prevention, the rates of hospitalization and acute mortality reached never seen magnitudes, and there was a shortage of protective equipment, such as PPE masks. This placed an overwhelming patient load on the health care system, and efforts to isolate the infected from the non-infected cases led to the complete reorganization of the hospital system. In addition, there was a shortage of health care providers both in acute and COVID care units, leading to the redirection of the workforce, coercing many to perform tasks that lied beyond their competencies. A number of health care workers became infected, some died, and many others had severe symptoms needing hospitalization and resulting in chronic post-COVID symptoms. There are no official statistics on the mortality rate among Hungarian health care workers related to the pandemic, however the Hungarian Medical Chamber published a list on its website in memory of the health care professionals whose death could be attributed to the COVID-19 infection based on the media and on various personal information sources (45).

During the fourth and fifth waves of COVID-19 (2021 September-December; 2022 January-May, respectively), vaccines were already available, and mortality associated with the omicron variant was lower; thus, we know from practical experience that mental health professionals’ fear of infection decreased.

Another unexpected finding was regarding the professional groups: only among psychiatrists did we find that their participation in COVID care was associated with an increase in stress and burnout scores. Among studies focusing on mental health professionals’ mental health, to our knowledge, this is the first that compares different subgroups working in similar settings and compares those who were involved in COVID care and those who were not. In a Croatian study (27), comparing psychiatrists with other specialists, they found that physicians working in other specialties had higher anxiety scores on the COVID-19 Anxiety Scale (CAS), but psychiatrists were also more prone to abuse drugs and sedatives compared to other specialists (the frequency of using sedatives was 1.6% among physicians and 2.8% among psychiatrists). Our study focused mainly on doctors and nurses working in psychiatric care in Hungary. Psychologists were affected by the pandemic in a very different way: while some of them also performed nursing tasks in COVID care, others worked in private practice (as well); hence, a more complex approach and survey would have been required to examine them in the various roles they played. Studies concerning psychologists and therapists have chiefly focused on the practice of teleconsultation and support for health workers (12, 46, 47), whereas in our study 31.9% of psychologists (15 persons) reported direct ‘bedside’ involvement in COVID care, a grouping of psychologists absent in relevant literature. No significant differences were found between psychologists’ stress and burnout scores according to whether they worked in COVID care or not. The low number of psychologist participants also makes us cautious about comparing data.

Furthermore, our study showed that for those on lower levels of the hierarchy (e.g., doctors in training, nurses), there was no significant difference in stress and burnout when comparing persons working in COVID care and those who are not. Their options and choices are basically limited by their professional position. We also know from experience that the competencies, the scope of duties, and the autonomy of psychiatrists working at the bedside of COVID patients changed significantly during the pandemic, and that psychiatrists had to perform a number of somatic medicine related tasks. COVID care was difficult for the specialists, psychiatry residents, nurses, and other staff involved, but the responsibility of making decisions rested primarily on the shoulders of specialists. We know that in practice there have been psychiatric patients who, because of their underlying psychiatric illness (e.g., paranoid schizophrenia) and somatic complaints (e.g., shortness of breath, weakness), were admitted to psychiatric wards that had been converted into COVID care units. However, there were also psychiatric patients who were admitted with mild symptoms, which became more severe in the course of their illness and required somatic care (e.g., monitoring of vital signs, administration of oxygen or specific drugs). In the presence or threat of respiratory complaints (e.g., alcohol-induced delirium), psychiatrists also had to carefully consider the provision of certain psychiatric drugs (e.g., benzodiazepines) to patients due to their respiratory depressant effects. These professional decisions and responsibilities may have been difficult for psychiatric specialists, and the current epidemiological situation may have induced chronic uncertainty, leading to a perceived loss of control.

While previous review articles have concluded that being a nurse during the pandemic is a risk factor for mental health problems (1, 44), our results showed no significant difference in stress and burnout between nurses and other mental health professionals. In our study, stress and burnout scores for each professional group (see **Table 3**) exhibited similar values, which may have been due to unpredictability and insecurity affecting all professions (secondments were necessary regardless of professional status). Of our sample of psychiatric workers, 58.2% was directly involved in COVID care. According to another Hungarian study (7), unpredictability was higher among non-physician health care workers compared to physicians, but no significant difference was found between physicians and non-physicians in terms of stress and burnout.

Prior studies conducted during the pandemic have emphasized that being a physician is a risk factor for burnout (e.g., 21, 48, 49). According to Li (21, 50), nurses consider their work a development opportunity, which has a protective effect against burnout. Lasalvia et al. (51, 52) state that the burnout of psychiatrists in mental health teams is the highest, however, our study did not confirm this claim. A study from Saudi Arabia (53) investigated burnout among psychiatry residents during the pandemic, which described a lower prevalence (27.3%) compared to a pre-pandemic systematic review (33.7%) (54), which was explained by a reduction in their duties during the outbreak. According to an Italian study among health workers, psychologists also experienced excessive work stress and burnout during the pandemic (55, 56), while another study states that psychologists who are effective in helping others are less effective in taking care of themselves and need to remedy this (57).

Regarding sleep quality, in our study, nurses scored significantly higher on the sleeping troubles subscale than psychiatrists, psychiatry residents, and psychologists. We explain this with the well-known negative effect of shift work on sleep quality. However, the literature again is divided on this subject: according to Cabeza et al.’s study in Columbia, during the first waves of the COVID-19 pandemic, psychiatrists and psychologists had more sleeping troubles than nurses, albeit this difference was not significant (58). In the review and meta-analysis of Salari et al. (59), the prevalence of sleep disorders during COVID-19 was higher among physicians as well. An African study (60) asserted that 23.9% of psychiatric workers were affected by sleep problems but found no significant association with professional position.

Our study also showed that participation in COVID care alone did not cause stress or burnout. According to Pappa et al. (61), concerns about the impact of COVID-19 on society and feeling pressured and uncomfortable at work contributed to burnout among mental health workers. Congruently, Rapisarda et al. (48) states that close contact with COVID patients also constituted a risk factor for burnout. A new study asserts it is unlikely that COVID-19 has caused as much damage to the mental health of most people as previous research has suggested. This systematic review examined the ‘general mental health’ factor before and during COVID in the general population; no changes in terms of mental health were found based on 94,411 unique titles and abstracts including 137 unique studies from 134 cohorts (62).

In our study, competence transgression had a moderating role only for stress, but not for burnout. Kagan et al. (63) examined mental health nurses/nurse managers, claiming many suffered from burnout and were overwhelmed by their new tasks and responsibilities, yet experienced high levels of satisfaction with their managerial performance because they viewed the extension of their nursing competence as a positive outcome (63, 64).

Workplace commitment was also found to have a moderating and negative effect on both stress and burnout; that is, the more committed one is to their job, the less stress or burnout one experiences. Among health care workers, stress and burnout have been described as negatively influencing the development of organizational commitment (65, 66;, 67, 68). It has also been reported that among nurses, professional competence did not show any effect on the development of organizational commitment (68, 69).

Emotional demands and work-family conflict had a moderating role in both stress and burnout. Martinez et al., who also worked with a version of the COPSOQ questionnaire during the first wave of COVID, described that health care workers reported worse health outcomes and higher exposure to psychosocial risks than the general salaried population. In addition to the high work demands and work pace, emotional demands were also high in health care workers, and frontline workers were more exposed to those psychosocial risks (70).

In our study, influence at work was selected out as a non-significant variable using the stepwise method. We also know from the pre-pandemic literature that for psychiatrists and mental health professionals, professional autonomy is essential for their mental health, job satisfaction, and for avoiding burnout (71–73). Wu’s study found (74) that low burnout scores among COVID care workers were associated with greater professional control and access to more information. Ogütlü et al. (75) conducted research among Turkish psychiatrists, most of whom reported moderate or high levels of stress related to the COVID-19 pandemic, and the majority of them also experienced moderate or high levels of work- and patient-related burnout, as well as lower levels of personal burnout. The latter was explained by the respondents’ confidence in their ability to manage the COVID-19 crisis themselves, which, according to the authors, indicates personal resilience and an internal locus of control.

Our results are in accordance with other findings stating that work-family conflict is related to burnout and stress. Kameg et al. surveyed mental health nurses (76), 64% of whom reported that the demands of their job often disrupted their family life; overall burnout scores remained moderate. This was explained by the fact that participants were generally able to cope effectively with the demands of the job, thus reducing burnout. Family life was a protective factor for caregivers during the COVID-19 pandemic, and family was an external source of support that mitigated burnout (77).

### Strengths

While there have been many studies on burnout and psychosocial factors among health care workers during the pandemic, we found relatively few studies that focused on mental health professionals who were directly involved in COVID care. We compared the psychosocial risk profile of 286 mental health professionals involved in and those not involved in COVID care at psychiatry units in various regions of Hungary. We compared data from team members with different professional experiences and roles (psychiatrist, psychiatry resident, nurse, and psychologist). We explored a wide range of work-related psychosocial risk factors in the context of COVID care. We collected data in the later phases of the COVID-19 pandemic, when the vaccine was already available and there was no strict lockdown, while most studies focused on the first waves and the initial crisis around the onset of the pandemic. We collected data from a relatively large sample of psychiatrists and psychiatry residents, a professional subgroup who took on special responsibilities during the pandemic. All age groups were well represented.

### Limitations

Our cross-sectional study supplied a snapshot of the psychosocial factors of mental health professionals, however, this research design provided no opportunity to explore longitudinal, causal relationships. Our questionnaire was aimed at a wide range of professionals working within psychiatric-psychotherapeutic care in Hungary, yet, because the number of respondents was relatively low, especially in some rural regions our survey cannot be considered representative. On the other hand, we think that our results can be generalized, as our sample included a similar number of respondents from various centers from the capital and from smaller settlements, and we found no regional differences in the main outcome variables. The online survey was active over a 5 month period, as data collection went less efficiently as we hoped. During this period, COVID was active and there was no change in the regulation of COVID care or in the functioning of psychiatric care units. Although we sent several reminders via our previously used recruitment channels, we had to close the survey at a lower - yet still acceptable - sample size. Based on personal information, the most common reasons for not completing the questionnaire were “we are already too busy and overwhelmed” and “I am not interested in any surveys”. We can draw limited conclusions regarding the specific stress of the psychologists, as some of them provided teleconsultations and others were directly involved in COVID care “at the bedside”, or both. Another limitation of the study is that we evaluated stress and burnout based on COPSOQ II questionnaire subscales, which did not allow us to examine the individual components of burnout.

## Conclusion

One important message of the study is that participation in COVID care was not a risk factor for stress and burnout per se, but that certain workplace factors played a pronounced role (e.g., work-family conflict, emotional demands). Not only risk factors, but also protective factors, such as workplace commitment, merit further investigation. Rethinking competences and setting up competence lists could also be a protective factor against stress.

It is important for psychiatrists to assess burnout and intervene. The working conditions of psychiatric nurses need to be rethought, with a particular focus on their mental health. Further research on stress and burnout among psychiatric workers is needed.

In many ways, COVID care has superseded previous forms of psychiatric care, such as rehabilitation care and teams; it is worthwhile to incorporate these workplace experiences into the transformation of evolving psychiatric care, for example, in terms of rethinking competencies.

We hope our research will provide a good foundation for further international comparative studies focusing on health care workers and mental health professionals.

## Data availability statement

The raw data supporting the conclusions of this article will be made available by the authors, without undue reservation.

## Author contributions

LM: Conceptualization, Data curation, Formal analysis, Investigation, Methodology, Resources, Writing – original draft. ÁZ: Conceptualization, Supervision, Writing – review & editing. AS: Conceptualization, Data curation, Formal analysis, Methodology, Resources, Supervision, Writing – original draft.
